# Circulating Interleukin-37 Levels in Healthy Adult Humans – Establishing a Reference Range

**DOI:** 10.3389/fimmu.2021.708425

**Published:** 2021-07-23

**Authors:** Danielle M. Santarelli, Fabien B. Vincent, Ina Rudloff, Claudia A. Nold-Petry, Marcel F. Nold, Marc A. Russo

**Affiliations:** ^1^ Genesis Research Services, Broadmeadow, NSW, Australia; ^2^ Centre for Inflammatory Diseases, School of Clinical Sciences at Monash Health, Monash University, Clayton, VIC, Australia; ^3^ Ritchie Centre, Hudson Institute of Medical Research, Clayton, VIC, Australia; ^4^ Department of Paediatrics, Monash University, Clayton, VIC, Australia; ^5^ Monash Newborn, Monash Children’s Hospital, Melbourne, VIC, Australia; ^6^ Hunter Pain Specialists, Broadmeadow, NSW, Australia

**Keywords:** biomarker, healthy, interleukin-37, plasma, reference range, serum

## Abstract

Interleukin (IL)-37 has an important function in limiting excessive inflammation. Its expression is increased in numerous inflammatory and autoimmune conditions and correlates with disease activity, suggesting it could have potential as a disease biomarker. Nevertheless, a reference range has yet to be determined. Our aim was to establish the first reference range of circulating IL-37 levels in healthy adult humans. PubMed was searched for studies reporting blood IL-37 concentrations in healthy adult subjects as measured by enzyme-linked immunosorbent assay. Nineteen studies were included in the analysis. Mean IL-37 levels were weighted by sample sizes, and weighted mean lower and upper levels ( ± 2SD of means) were calculated to provide a weighted mean and reference range. IL-37 levels were quantified in either serum or plasma from a total of 1035 (647 serum; 388 plasma) healthy subjects. The serum, plasma and combined matrix weighted means (reference ranges) were 72.9 (41.5 – 104.4) pg/mL, 83.9 (41.1 – 126.8) pg/mL, and 77.1 (41.4 – 112.8) pg/mL, respectively. There were no significant differences between serum and plasma means and upper and lower limits. Study means and upper IL-37 levels were significantly higher in Chinese population studies. From our analysis, a preliminary reference range for circulating IL-37 levels in healthy human adults has been established. In order to determine a reliable reference range for clinical application, large, prospective, multi-ethnic, healthy population studies are necessary. In addition, demographics, sample matrix, collection, processing and storage methods potentially affecting IL-37 detection levels should be thoroughly investigated.

## Introduction

**Figure 1:**
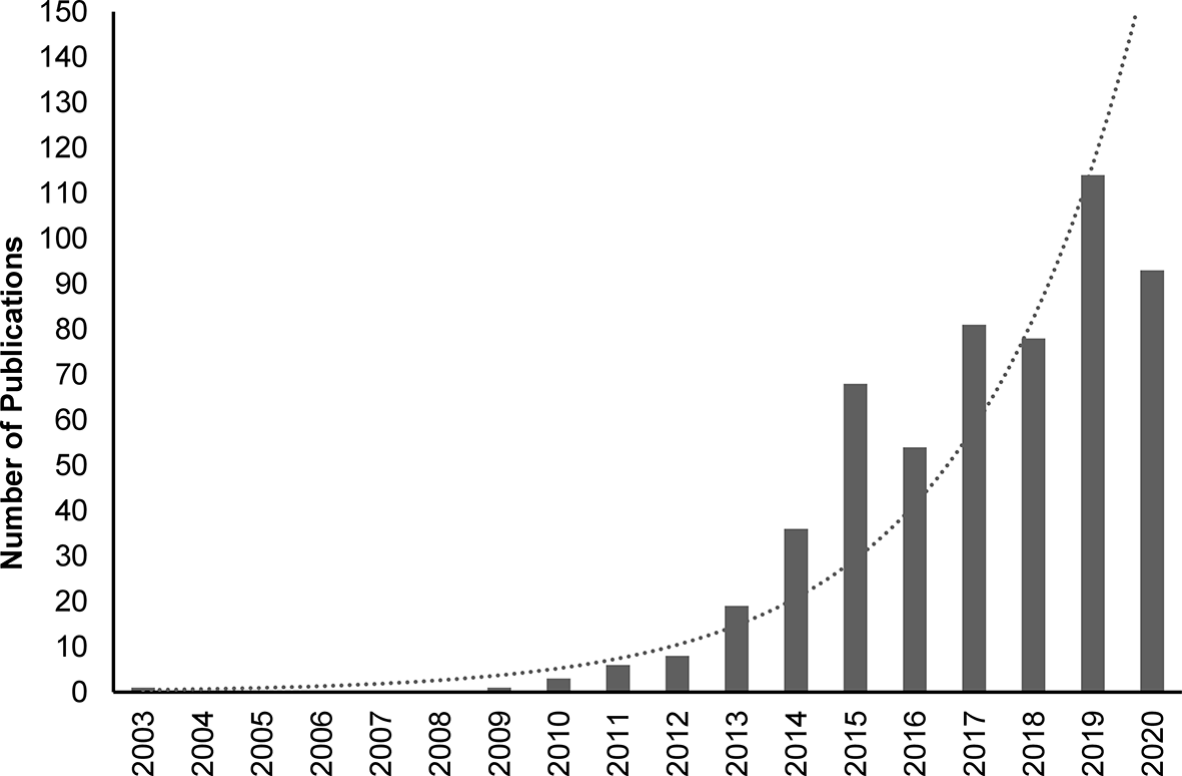
PubMed-indexed peer-reviewed publications on IL-37 between 2003 and 2020. Search was performed on 12^th^ January 2021 using the following search terms: IL37, IL-37, IL 37, Interleukin-37, Interleukin37, Interleukin 37. Dotted line: exponential trendline.

Interleukin (IL)-37, formally known as IL-1 family member 7 (IL-1F7), is the second most recent addition to the IL-1 family (1–3). Unlike most IL-1 family members, which are pro-inflammatory, IL-37 has anti-inflammatory actions (4). Research over the last decade has provided detailed insights into the fundamental mechanisms of action of IL-37, and highlighted its key role in suppressing innate immune and inflammatory responses (3–6). The importance of IL-37 as an anti-inflammatory cytokine (7) is highlighted by the exponentially increasing number of peer-reviewed publications dealing with this cytokine (**Figure 1**).

The primary sources of IL-37 are circulating innate immune cells, including monocytes, dendritic cells and macrophages (8, 9). Under healthy conditions, IL-37 protein expression levels are low, as an mRNA instability sequence has been identified in the IL-37 coding region and therefore IL-37 mRNA is modulated under normal conditions (10). In peripheral blood mononuclear cells (PBMCs), IL-37 expression is upregulated by inflammatory stimuli such as IL-1ß, IL-18, tumor necrosis factor (TNF), interferon gamma (IFNγ), and toll-like receptor (TLR) ligands (3, 11, 12). In turn, IL-37 is a potent inhibitor of pro-inflammatory cytokine production, in particular, but not limited to, TNF, IL-1ß and IL-6 (6). Thus, IL-37 likely acts as a negative feedback mechanism to limit excessive inflammation (3, 13). Additionally, IL-37 acts to restore cell metabolic homeostasis during inflammation and to limit the metabolic cost of chronic inflammation (6, 13, 14). One mechanism by which IL-37 suppresses pro-inflammatory cytokine production is by “switching off” metabolic activation in monocytes (13).

Five distinct isoforms of IL-37 (a-e) have been identified, each with tissue-specific expression patterns and different biological functionality (11). Of the five, IL-37b (exons 1, 2, 4, 5 and 6) is the longest and the most biologically active isoform and has been the one mostly studied (7). Exon 1 is included in all isoforms except IL-37a, which still appears to share similar anti-inflammatory effects to IL-37b, while IL-37c and IL-37e are considered to act as regulators of IL-37a and IL-37b (7). Less is known about IL-37d, which also presents with anti-inflammatory functions and is detected in PBMCs (15). Of note, in the literature, the IL-37b isoform is often referred to as “IL-37”. Currently, there have been a total of 14 IL-37 protein variants identified, with three major variants constituting over 97% of all sequences, including “Var1”, “Var2” and “Ref” (16). Within the European population, 13.5% are heterozygous for the third major variant, “Var2”, which, compared to the two other major variants, induces a stronger, yet shorter-lived immune response due to preferential proteasome degradation (17).

Since the discovery of IL-37’s potent anti-inflammatory function, research into its role in disease pathogenesis has been growing. Compared to healthy subjects, increased circulating (serum and plasma) levels of IL-37 have been documented in various inflammatory and autoimmune conditions, including rheumatoid arthritis (18–24), ankylosing spondylitis (25), endometriosis (26–28), systemic lupus erythematosus (29–31), Guillain-Barré syndrome (32), Graves’ disease (33), and multiple sclerosis (34, 35), as well as in spinal cord injury (36) where its levels are frequently correlated with measures of disease activity. Increased IL-37 is likely one of the body’s attempts to dampen inflammation and restore immune homeostasis. Elevated circulating IL-37 levels have also been reported in several types of cancers (e.g. epithelial ovarian cancer, gastric cancer), and chronic heart failure, and were associated with poor prognosis (37–39). Conversely, decreased circulating IL-37 abundance has been observed in some inflammatory diseases, such as Behçet’s disease (40, 41) and inflammatory bowel disease (ulcerative colitis and Crohn’s disease) (42). Interestingly, in patients with inflammatory bowel disease, increased local abundance has been reported in colonic/intestinal tissues (42–44). IL-37 protein level has also been studied in other body fluids of patients suffering from inflammatory diseases, including the cerebrospinal fluid of patients with Behçet’s disease with neurological localizations (lower IL-37 levels) (45), and in those with Guillain-Barré syndrome (higher IL-37 levels) (32). A growing number of cell line studies, animal model studies, and genetic studies provide further support for involvement of IL-37 in the aforementioned and additional conditions, such as gout, in which rare genetic variants of IL-37 that result in loss of anti-inflammatory action have been identified (46), obesity-induced inflammation and metabolic syndrome (47, 48), melanoma (49), and other cancers (50).

The potential use of IL-37 as a disease biomarker is often discussed, however, such an approach would require a reference range to be established, and to the best of our knowledge, no attempts have been made yet. Importantly, bearing in mind low physiological levels of IL-37 in the circulation, while differential expression of IL-37 is reported in many inflammatory diseases compared to healthy controls, the direction of change appears inconsistent across diseases, highlighting the need for hyper sensitive assays that can determine both low and high thresholds for normal ranges. Additionally, the dynamics and magnitude of expected fluctuation of IL-37 levels over the course of each disease should be studied.

Limited reports have focused on establishing reference ranges or cut-off levels for cytokines to develop robust diagnostic or prognostic biomarkers (51). Kleiner et al. (52) recognized the unmet need for reference values to be established in physiological conditions and investigated the levels of 48 cytokines and chemokines in the serum of 72 healthy subjects, however, IL-37 was not included in their analysis. Most human cytokine biomarker studies primarily aim to identify proteins with the largest significant difference in their levels between the cohort of patients compared to healthy subjects, and to find relationships between candidate biomarkers and disease phenotype, activity, or severity. The clinical translation of such findings into new diagnostic or prognostic tools is difficult and also limited due to the lack of cytokine reference ranges or consensus cut-offs.

This study aimed to review the existing literature on circulating levels of IL-37 in healthy adult subjects and to derive a preliminary reference range using published meta-analysis-type methods (53–55).

## Methods

### Search Strategy

A Medline search was performed *via* PubMed for peer-reviewed articles published up to the 1st March 2021 that reported on blood IL-37 concentrations in healthy humans using the following search term: (il37 OR il-37 OR “IL 37” OR interleukin37 OR interleukin-37 OR “interleukin 37”) AND (healthy) AND (serum OR plasma OR blood). Search results were filtered for English language.

### Study Selection

Full articles were reviewed and screened. For inclusion into the analysis, studies had to report serum or plasma levels of IL-37 from healthy adults as measured using enzyme-linked immunosorbent assays (ELISA). IL-37 levels had to be reported as mean + standard deviation (SD) (or standard error of the mean).

**Figure 2:**
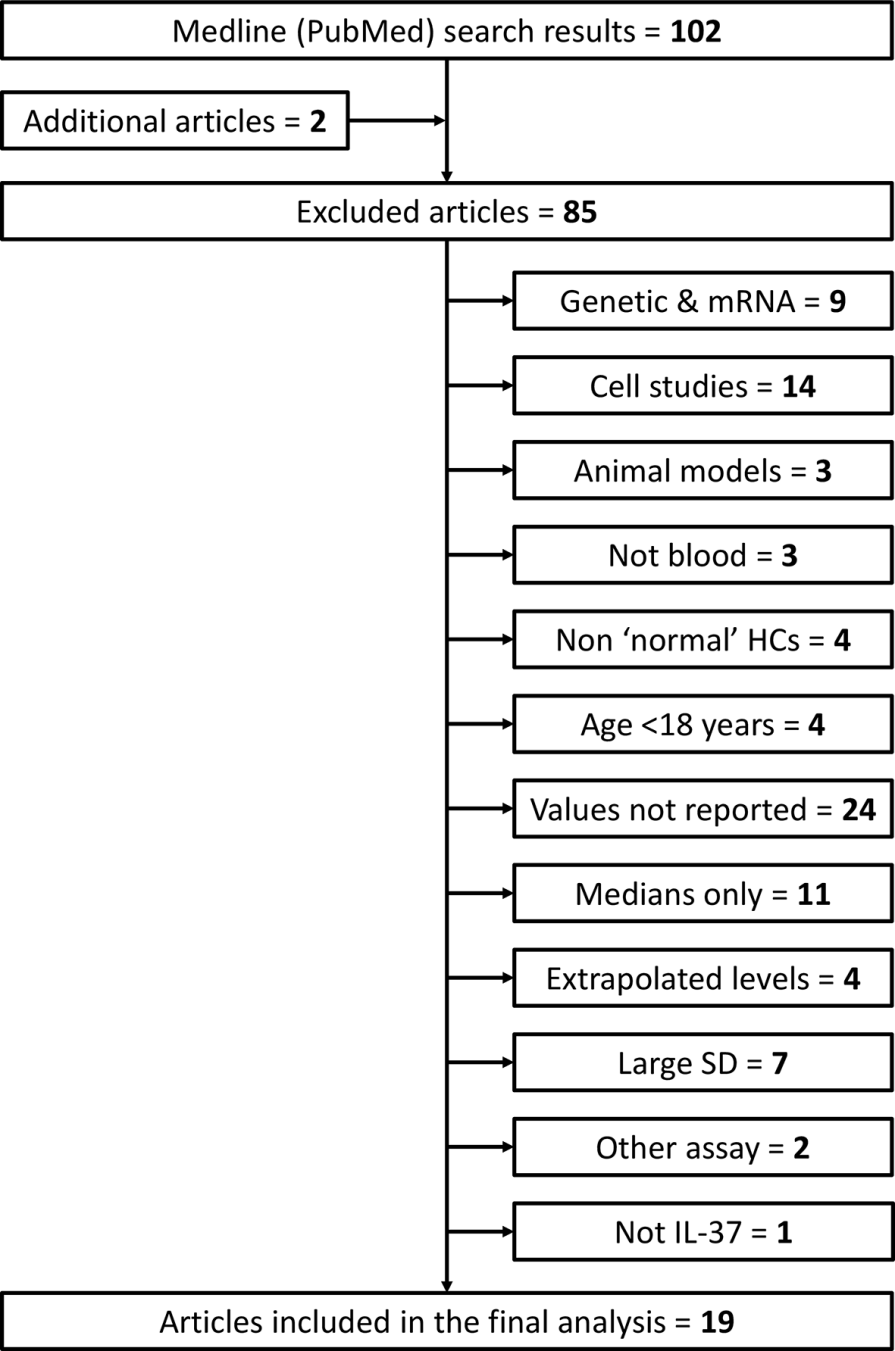
Flow diagram of search results and article screening, exclusion and inclusion for analysis of circulating IL-37 in healthy adult humans. Additional articles were found by reviewing the ‘Similar Articles’ listed on abstract pages on PubMed. HCs, healthy controls; SD, standard deviation.

Studies were excluded if they met any of the following exclusion criteria: 1) only genetic or gene expression (mRNA) studies; 2) cell/tissue studies; 3) animal model studies; 4) IL-37 levels measured in non-blood matrix (e.g., urine, saliva, sputum); 5) healthy control subjects considered non ‘normal’ (e.g., pregnant females or other patients); 6) healthy subjects < 18 years of age; 7) numerical values of mean (SD) circulating IL-37 not reported/available (e.g., graphical results only); 8) only median IL-37 levels reported; 9) extrapolated IL-37 levels reported (outside of the detection limits of the ELISA kit used); 10) large standard deviations that result in calculation of negative lower limit values; 11) quantification by method other than ELISA; 12) not IL-37 (misleading article title). Additional articles listed under ‘Similar Articles’ on the article abstract pages in PubMed were also reviewed. This screening process is summarized in **Figure 2**.

### Data Collection

The following healthy subject data was extracted: mean (SD) IL-37 blood concentration in pg/mL, sample type (serum or plasma), number of healthy subjects; and (if available): mean age (SD or range), gender frequencies. ELISA quantification kit manufacturer and the country in which the study was performed was also recorded.

### Statistical Analysis

All data analyses and data visualization were performed within Microsoft Excel 2016 (Version 2102) using spreadsheets developed by Neyeloff et al. (56) for conducting meta-analyses. To calculate a reference range for circulating IL-37 in healthy adult subjects, we considered the approaches taken by Nemeth et al. (55) and Venner et al. (53) to calculate references ranges for other molecular biomarkers, and applied the methodology of Thijs et al. (54) and Staessen et al. (57, 58) to determine reference values for blood pressure.

To calculate a reference value (mean) for IL-37 levels in healthy adults, the mean of each included study (*x_i_*) was weighted by its respective sample size (*n_i_*) and the sum of the weighted means was then divided by the sum of the sample sizes to calculate a weighted mean (*M*) as follows:

$$\text{M}=\frac{\Sigma_{\text{i}=1}^{\text{n}}{\text{n}}_{\text{i}}{\text{x}}_{\text{i}}}{\Sigma_{\text{i}}^{n}{\text{n}}_{\text{i}}}$$

To calculate a reference range, lower and upper limits of normal IL-37 levels were first calculated for each included study as mean ± 2 SD, and the same formula was then applied to calculate a weighted mean lower value and a weighted mean upper value. The weighted means and reference range for serum, plasma, and all studies combined are presented. A forest plot was generated using the methods and spreadsheet developed by Neyeloff et al. (56).

## Results

### Study Characteristics

After screening 102 articles returned by the initial PubMed search (+ additional “Similar” articles), 19 met the inclusion criteria (**Figure 2**). Twelve and seven studies measured IL-37 levels in serum and plasma, respectively. The total number of subjects included in the meta-analysis was 1035 (serum: n = 647; plasma: n = 388). Study characteristics and healthy subject demographics are presented in **Table 1**.

**Table 1:** Description of studies included in the analysis, with healthy adult subject demographics, study-reported mean IL-37 levels, and computed healthy IL-37 level ranges.

Study	Matrix	n	Mean/*Median* (SD/*Range*) Age (years)	Gender (M | F)	Country	ELISA Kit Manufacturer	Mean (SD) IL-37 (pg/mL)	Lower Range (pg/mL)	Upper Range (pg/mL)
Zhou et al. (59)	Serum	102	18.9 (16.1)	53 | 49	China	Adipogen	129.2 (19.2)	90.7	167.7
Huo et al. (37)	Serum	76	*52 (26–64)*	0 | 76	China	Adipogen	84.9 (28.9)	27.1	142.7
Kouchaki et al. (34)	Serum	75	31.8 (11.8)	15 | 69	Iran	MyBioSource	36.6 (15.2)	6.1	67.1
Abushouk et al., (60)	Serum	70	*25 (12-70)*	56 | 14	Sudan	Abcam	22.1 (2.4)	17.3	26.8
Chen et al. (36)	Serum	52	49.4 (5.2)	31 | 21	China	Abcam	108.6 (11.7)	85.0	131.9
Tawfik et al. (30)	Serum	50	29.7 (4.3)	7 | 43	Egypt	Elabscience	44.5 (16.9)	10.7	78.4
Farrokhi et al. (35)	Serum	49	35.3 (8.0)	30 | 19	Iran	eBioscience	114.6 (20.6)	73.5	155.8
Sehat et al. (61)	Serum	47	30.2 (4.8)	18 | 29	Iran	MyBioSource	41.1 (15.1)	10.8	71.3
Song et al. (19)	Serum	46	44.7 (13.7)	17 | 29	China	Invitrogen	114.2 (22.5)	69.3	159.1
Wang et al. (62)	Serum	30	65.9 (12.2)	13 | 17	China	Unknown	23.8 (2.5)	18.7	28.8
Yildiz et al. (63)	Serum	30	43.8 (7.6)	–	Turkey	Bioassay Technology Laboratory	13.7 (4.7)	4.3	23.1
Kaabachi et al. (26)	Serum	20	30.9 (8.0)	0 | 20	Tunisia	Genway Biotech	74.1 (13.5)	47.1	101.1
Ding et al. (64)	Plasma	112	42.9 (12.5)	55 | 57	China	R&D Systems	47.6 (5.9)	35.8	59.5
Yang et al. (18)	Plasma	100	52 (14.1)	60 | 40	China	Adipogen	84.6 (36.8)	11.0	158.2
Wang et al. (65)	Plasma	56	56.5 (16.5)	40 | 16	China	Adipogen	120.6 (20.0)	80.6	160.6
Zhang et al. (66)	Plasma	45	*68 (57–72)*	24 | 21	China	Elabscience	111.2 (36.2)	38.8	183.7
Li et al. (67)	Plasma	30	*65 (42–84)*	17 | 13	China	R&D Systems	150.4 (15.5)	119.5	181.3
Shou et al. (38)	Plasma	30	–	–	China	Adipogen	45.2 (11.6)	22.1	68.3
Zhang et al. (68)	Plasma	15	*40 (16-66)*	6 | 9	China	Adipogen	75.6 (27.5)	20.6	130.7

ELISA, enzyme-linked immunosorbent assay; F, female; M, male; n, number of healthy adult subjects; SD, standard deviation.

Lower Range = mean – 2 SD; Upper Range = mean + 2 SD.

### Circulating IL-37 Reference Ranges in Healthy Adults

**Figure 3:**
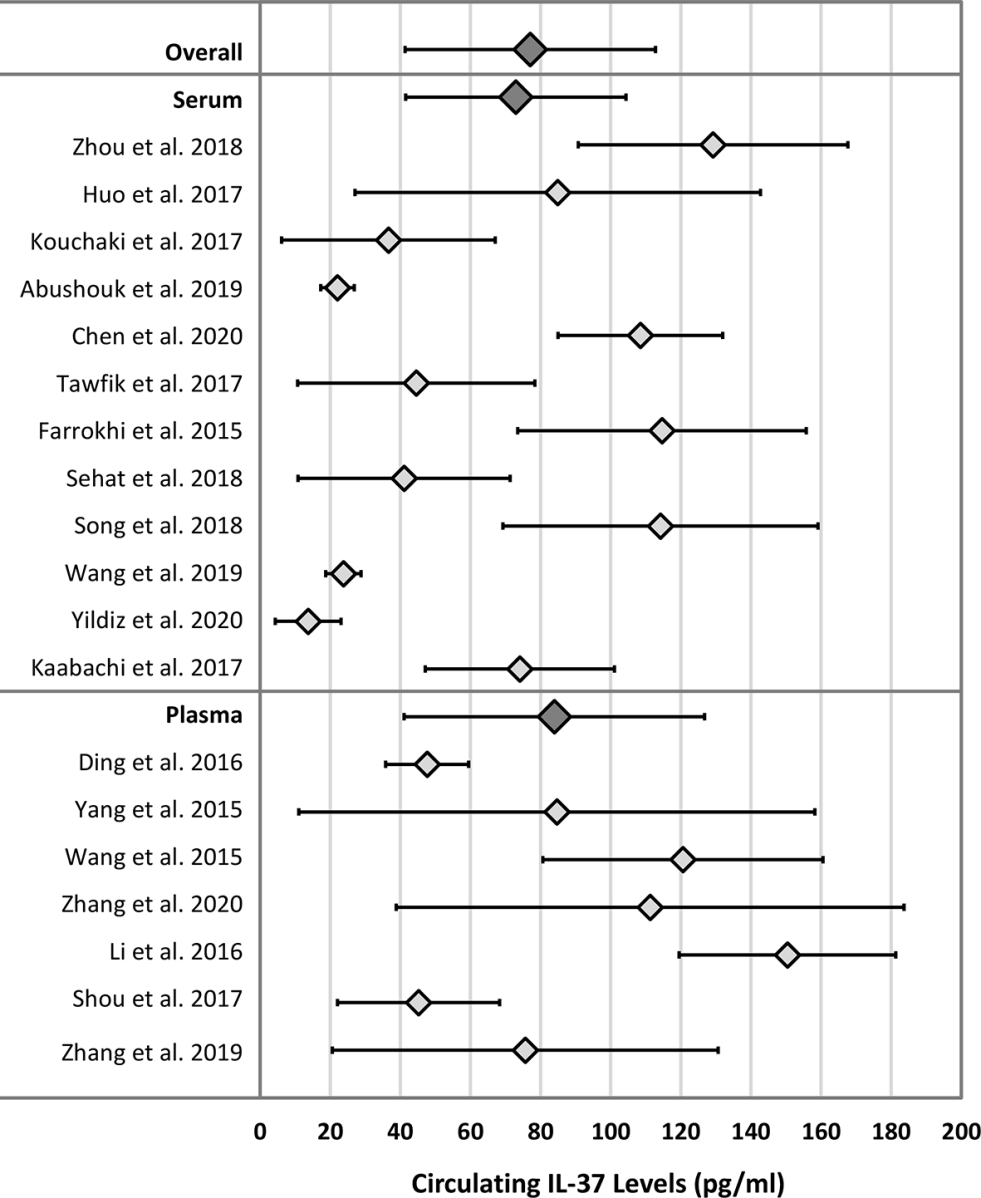
Forest plot of IL-37 means and ranges (± 2 SD) for individual studies and mean of weighted means and reference ranges. Dark grey diamonds: Overall - mean of weighted means and reference range of IL-37 in both serum and plasma matrix; Serum - mean of weighted means and reference range of IL-37 in serum only; Plasma - mean of weighted means and reference range of IL-37 in plasma only.

The weighted means (reference ranges) for serum and plasma IL-37 in healthy adult subjects were 72.9 (41.5 – 104.4) pg/mL and 83.9 (41.1 – 126.8) pg/mL, respectively. The pooled (serum and plasma) weighted mean (reference range) of IL-37 in healthy adult subjects was 77.1 (41.4 – 112.8) pg/mL. Results are summarized in **Table 2**. **Figure 3** displays a forest plot of individual study mean (± 2 SD) IL-37 levels, and the weighted means (reference ranges) for serum, plasma, and pooled serum and plasma IL-37 levels. No statistically significant difference in weighted means, and lower and upper range IL-37 levels were observed between serum and plasma groups (*P* = 0.24, *P* = 0.62, *P* = 0.15, respectively; unpaired two-tailed *t*-test).

**Table 2:** Circulating IL-37 weighted means and reference ranges for subgroups analyzed.

Group	Weighted Mean (pg/mL)	Reference Range (pg/mL)
Pooled (n = 1035)	77.1	41.4 – 112.8
**Matrix**		
Serum (n = 647)	72.9	41.5 – 104.4
Plasma (n = 388)	83.9	41.1 – 126.8
**Country**		
China (n = 694)	91.9	51.0 – 132.8
Other (n = 341)	46.8*	21.7 – 71.9*

n, total healthy adult subjects.

*Statistically significant different means and upper levels between Chinese and non-Chinese population studies.

Twelve of the 19 included studies involved Chinese populations (mixed plasma and serum studies), with a weighted mean (reference range) of IL-37 of 91.9 (51.0 – 132.8) pg/mL. When compared to the non-Chinese population (serum studies only), for which the weighted mean (reference range) of IL-37 was 46.8 (21.7 – 71.9) pg/mL, mean and upper range IL-37 levels were statistically significantly higher in the Chinese populations (*P* = 0.03 and *P* = 0.03, respectively; unpaired two-tailed *t*-test). Results are summarized in **Table 2**.

There were two female population studies (endometriosis and epithelial ovarian cancer investigations), and two studies with > 85% female healthy adult subjects. For these four studies, the weighted mean (reference range) was 58.4 (18.1 – 98.7) pg/mL. No statistically significant difference in mean, lower and upper range of IL-37 levels were observed between these four studies (total 208 females and 22 males; all serum studies) and the remaining 13 studies with male and female counts reported (total 334 females and 420 males; mixed plasma and serum studies) (data not shown; *P* = 0.23, *P* = 0.15, *P* = 0.39, respectively; unpaired two-tailed *t*-test).

It is worth noting that the most commonly used ELISA kit was manufactured by AdipoGen (n = 6). The weighted mean IL-37 level for healthy subjects from studies using this kit was 98.5 pg/mL, with study means ranging from 45.2 to 120.6 pg/mL for plasma (n = 4), and 84.9 to 129.2 pg/mL for serum (n = 2) (data not shown). The remainder of the included studies used human IL-37 ELISA kits manufactured by Abcam, Bioassay Technology Laboratory, eBioSource, elabscience, GenWay, Invitrogen, MyBioSource, and R&D Systems (**Table 1**).

## Discussion

IL-37 has recently emerged as a critical regulator of inflammation. The growing number of studies on inflammatory and autoimmune diseases that have reported significant differences in circulating IL-37 levels between patients and control subjects highlights the importance of this cytokine in disease settings, and supports its potential widespread usefulness as a disease biomarker. Additionally, numerous reports of associations between circulating IL-37 levels and inflammatory pathology parameters (i.e.: C-reactive protein, CRP; erythrocyte sedimentation rate, ESR) support the potential usefulness of circulating IL-37 levels as a biomarker of disease activity or severity. Circulating IL-37 levels have been found to positively correlate with levels of CRP and/or ESR in patients with rheumatoid arthritis (18, 21–23), systemic lupus erythematosus (30), adult-onset Still’s disease (69, 70), ankylosing spondylitis (25), gout (71), and osteoarthritis (72), as well as in patients with chronic heart failure (38). Also, the prognostic value of IL-37 has been demonstrated for several diseases, including epithelial ovarian cancer (37), gastric cancer (39), multiple sclerosis (34), spinal cord injury (36), acute ischemic stroke (66), and heart failure (38). In this respect, we have conducted a meta-analysis of 19 studies, analyzing a total of 1118 healthy adult individuals using extracted mean (SD) levels to establish, for the first time, a preliminary reference range of circulating IL-37 in both serum and plasma.

To the best of our knowledge, no published study has defined a reference range for circulating IL-37 levels in healthy individuals. Several studies have evaluated IL-37 as a disease biomarker, using receiver operating characteristic (ROC) curve analysis to assess the sensitivity and specificity of cut-off values of circulating IL-37 to distinguish patients from healthy subjects. Yuan et al. (73) reported a cut-off value of 253.86 pg/mL (plasma), with high sensitivity and specificity, to distinguish rheumatoid arthritis patients from controls. Yildiz et al. (63) reported a cut-off value 9.37 pg/mL (serum) to distinguish healthy subjects from patients with asthma, however, sensitivity was poor. For endometriosis, serum IL-37 cut-off levels of 69.84 pg/mL (28), and 5.99 pg/ml (27), both with high sensitivity and specificity, have been reported. Future research that aims to prospectively quantify IL-37 levels in serum and plasma in large, healthy populations would be of value.

According to the Clinical and Laboratory Standards Institute (CLSI) guidelines (74), each laboratory should determine the circulating levels of the analyte of interest in a minimum of 120 samples from healthy individuals for each major demographic group (e.g. age group, gender, ethnicity) for establishing optimal reference intervals. Only three of the 19 analyzed studies were of modest sample size, with n ≥ 100, yet none were ≥ 120. Ding et al. (64) conducted the largest study, including 112 healthy subjects, and reported a mean (SD) plasma IL-37 level of 47.6 (5.9) pg/mL, which computes to a range of 35.8 – 59.5 pg/mL. This range may be considered too narrow based on the results of this meta-analysis. Two other analyzed studies (60, 62) reported small SDs (apparently not standard error of the means, as indicated throughout each article), which also compute to narrow ranges. A number of excluded studies reported excessively large SDs that translated to negative lower limits. It would be valuable to know if any outliers were identified in these studies, and if sensitivity analyses were performed to assess the impact of the outliers on the study outcome.

As per the CLSI guidelines described above, it is important to consider inter- but also intra-individual variation when establishing a reference range. Blood levels of many cytokines are associated with different factors, including demographics (age, gender, ethnicity) and lifestyle factors, as shown in cross-sectional analyses (51, 52, 75). For example, Kleiner et al. (52) investigated the relationship between age and serum levels of 48 cytokines and chemokines (not including IL-37) in healthy subjects categorized by age groups (1-6, 7-17, and ≥ 18 years). They reported nine cytokines/chemokines that were significantly decreased and two that were significantly increased in adults compared to children. Of note, no significant differences between males and females across age groups were reported. Stowe et al. (75) investigated levels of IL-6, IL-10, and other cytokines, in a population-based study of Hispanic and non-Hispanic ethnic groups. They reported significantly higher levels of plasma IL-6 and TNF-r1 in subjects aged over 50 (entire cohort), and significantly higher levels of plasma IL-6 in the “non-Hispanic black” ethnic group compared to the “non-Hispanic white” ethnic group.

To our knowledge, circulating IL-37 (as measured by ELISA) has not been investigated in relation to age and ethnicity in a healthy population-based study setting. Only one of the studies included in our analysis investigated associations between gender and IL-37 levels in healthy subjects, showing no significant differences in serum IL-37 levels according to gender (35). Davarpanah et al. (76) (excluded study) reported the same findings. In light of the limited number of studies, a larger and independent healthy population study is warranted. Of note, there is limited evidence suggesting that IL-37 levels are negatively associated with age in infectious and autoimmune disease settings, including eumycetoma infection (60), Hashimoto’s thyroiditis (77), and multiple sclerosis (78). Increased serum IL-37 was also reported to be associated with Asian ethnicity in patients with systemic lupus erythematosus (79). In our analysis, we observed significantly higher mean of weighted means of IL-37 levels and calculated upper levels across the Chinese studies compared to non-Chinese population studies. These observations are, however, preliminary in light of the small number of studies in the non-Chinese group, and the potential for the matrix studied (plasma/serum) as a confounding factor. In fact, Chinese population studies included both serum (n = 5) and plasma (n = 7), while the non-Chinese studies included only serum investigations (n = 7) (**Table 1**). Future research is needed to confirm these preliminary findings in a prospective, large, and multi-ethnic cohort of healthy subjects.

Amongst the included studies, only cross-sectional analyses of healthy subjects were performed. Circulating levels of several other cytokines have also been shown to correlate with temporal factors such as stress, weight, diet, fasting status, and blood pressure (51, 80). In a 14-week study of cytokine serum levels (including 12 interleukins) in healthy subjects (using ultrasensitive single-molecule array assays), intra-individual variability across study visits was low and temporal stability was high for all cytokines, however, IL-37 was not measured (81). Longitudinal studies are therefore needed to investigate intra-individual variability in IL-37 levels in healthy adults.

There was a large degree of variation in circulating IL-37 levels between ELISA kits. In this regard, it is of importance that the performance of specific IL-37 detection tools always needs to be carefully validated by the user. Our team has therefore selected four different commercially available IL-37 ELISA kits for validation. PBS or murine serum spiked with recombinant IL-37 (recIL37) was used as a positive control. In every ELISA, positive controls were then tested alongside either murine serum samples from mice either injected with a bolus of recIL-37 or vehicle (negative control) (82), or systemic lupus erythematosus patient serum (79) to determine endogenous IL-37 levels. In our hands, the AdipoGen ELISA provided the most reliable results, detecting endogenous IL-37 in human serum or serum of mice injected with recombinant human IL-37. Importantly, the AdipoGen kit was also used in the majority of the studies included herein. For a comprehensive conclusion, a head-to-head comparison study of commercial ELISA kits in a broad selection of specimens would be required, which is beyond the scope of this study. Notably, our team has also extensively tested tools for the detection of IL-37 in other applications and found the IL-37 antibody clone 37D12 from eBioscience (San Diego, CA, USA) to reliably detect IL-37 by flow cytometry (8), immuno blot and immunohistochemistry (83).

Parameters other than the detection method by the kit per se, such as pre-analytics (sample collection, processing, storage) (84), assay procedure (room temperature, timing of analysis, plate reader) (85), the matrix studied (plasma *vs* serum), or study population heterogeneity (demographics, diet, alcohol consumption), are of importance and need to be considered as potential confounding factors. The complexity of comparing individual cytokine levels measured using different methods or platforms (including variability between assay kits from different manufacturers) has been discussed in detail by Tarrant (86), who focused on the use of blood cytokines as biomarkers of toxicity. Standardized operating procedures for pre-analytics (84) should be established by commercial kit manufacturers to optimize circulating IL-37 quantification. Sample collection and processing was poorly documented in several of the articles that we reviewed. Establishing a reference range for both serum and plasma samples would be valuable, as protein levels are known to differ according to the matrix (87). Whether this applies to IL-37 has yet to be determined. We observed no statistically significant difference in mean of weighted means, lower and upper range IL-37 levels between serum and plasma when included studies were grouped by matrix.

If circulating IL-37 is to be measured in clinical settings, then reference ranges should be established for each kit by the manufacturer, and also by the laboratory performing the test if the population tested differs from the one assessed by the manufacturer. However, it has been argued that this system does not serve the medical community well and that development of a common universal reference range is ideal (88). According to the CLSI guidelines (74), if a universal reference range that meets the criteria is established then an appropriate alternative would be for individual laboratories (and manufacturers) to verify the reference range by analyzing as little as 20 samples collected from healthy individuals recruited from the local population.

The major limitations of our study have already been highlighted. We acknowledge that the quality of the included studies potentially hinders the conclusions drawn from our analysis. Additionally, despite there being a large number of potentially useful studies in the literature, many were excluded as they did not report actual mean (SD) values for IL-37 levels in healthy subjects, presenting results graphically only, or reporting only the direction and significance level of any changes between groups. For studies that were excluded for reporting medians and minimum and maximum values only, we attempted to estimate means and standard deviations using methods by Luo et al. (89) and Wan et al. (90), however, meaningful estimations could not be achieved. Overall, this underlines the unmet need for any such study to report both mean (SD) and median (IQR) regardless of the cytokine data distribution in each of the studied groups, and to report the actual values in the text, even if the data are graphically presented. To enable meta-analysis to be performed in future research, such guidelines should be developed in collaboration with peer-reviewed journals.

Furthermore, the potential confounding effects of study population variables were not thoroughly assessed. Our aim was to provide a starting point for a reference range, the accuracy of which could be improved by performing a larger, prospective, multi-ethnic study of both serum and plasma IL-37 levels in healthy adult humans, including detailed documentation of demographic and lifestyle parameters for analysis as potential confounding factors in multivariable regression models.

The important role of IL-37 in suppressing innate immune and inflammatory responses and the growing mound of evidence for its involvement in various disease states warrants further investigations into what may be considered normal circulating levels in humans. Our study provides, for the first time, a preliminary reference range for circulating plasma and serum IL-37 levels. In order to establish a definitive reference range for both matrices, large, high-quality, prospective, longitudinal, multi-ethnic healthy population studies, including a balanced gender ratio as well as all age categories, that meet CLSI criteria, are needed.

## Data Availability Statement

The original contributions presented in the study are included in the article/supplementary material. Further inquiries can be directed to the corresponding author.

## Author Contributions

MAR conceptualized and designed the study. DMS collected and analyzed the data. MAR and DMS initiated the manuscript. All authors interpreted the data, contributed to the manuscript, revised the manuscript, and approved the submitted version.

## Funding

This work was supported by the National Health and Medical Research Council (NHMRC) of Australia Investigator Grant Leadership 1 [Grant 1173584] to CN-P, and the Fielding Foundation Fellowship 2017 to MN. FV is supported by a NHMRC Emerging Leadership 1 [Grant 1196112].

## Conflict of Interest

Monash University, Hudson Institute (CN-P and MN) hold two patent families on IL-37, namely PCT/AU2016/050495 and EP19218657.5. No other conflicts of interest exist for these authors.

The remaining authors declare that the research was conducted in the absence of any commercial or financial relationships that could be construed as a potential conflict of interest.

## Publisher’s Note

All claims expressed in this article are solely those of the authors and do not necessarily represent those of their affiliated organizations, or those of the publisher, the editors and the reviewers. Any product that may be evaluated in this article, or claim that may be made by its manufacturer, is not guaranteed or endorsed by the publisher.
